# Exploration of the relationship between sleep position and isolated tongue base or multilevel surgery in obstructive sleep apnea

**DOI:** 10.1007/s00405-012-1995-6

**Published:** 2012-03-20

**Authors:** J. P. van Maanen, M. J. L. Ravesloot, B. I. Witte, M. Grijseels, N. de Vries

**Affiliations:** 1 Department of Otolaryngology/Head Neck Surgery, Sint Lucas Andreas Ziekenhuis, Jan Tooropstraat 164, 1061 AE Amsterdam, The Netherlands; 2 Department of Epidemiology and Biostatistics, VU University Medical Centre, Amsterdam, The Netherlands; 3 Department of Otolaryngology/Head Neck Surgery, Leiden University Medical Centre, University of Amsterdam, Amsterdam, The Netherlands

**Keywords:** Obstructive sleep apnea, Sleep surgery, Posture

## Abstract

This study aimed to elucidate the role of sleep position as a confounding factor on apnea hypopnea index (AHI) and surgical success in isolated tongue base or multilevel surgery. This study was conducted using retrospective analysis of patients who underwent hyoid suspension because of obstructive sleep apnea (OSA), in the St. Lucas Andreas Hospital, Amsterdam, The Netherlands, from 2004 to 2011. Concurrent surgical treatment was documented. Sleep positions and corresponding AHIs before and after surgery were compared. A total of 130 patients were included. 94 patients underwent surgery of base of tongue and palate (either uvulopalatopharyngoplasty or Z-palatoplasty), of whom 72 underwent concurrent radiofrequent thermotherapy of the base of tongue. 36 patients underwent base of tongue surgery alone, of whom 22 underwent concurrent radiofrequent thermotherapy of the base of tongue. 65 patients either had a successful reduction in AHI or in AI. Isolated tongue base or multilevel surgery was as successful on the supine AHI as it was on the AHI in other sleeping positions. Surgery was not more successful in the group with position-dependent patients as compared with the non-position-dependent patients (*P* = 0.615). Successful and non-successful surgical results could not be explained by variations in percentages of supine sleep position. Sleep position is not a confounding factor on surgical outcomes in tongue base surgery. The results of isolated base of tongue or multilevel surgery in position-dependent OSA patients leave room for improvement, possibly through positional therapy.

## Introduction

Obstructive sleep apnea (OSA) is the most prevalent sleep-disordered breathing problem, affecting 2–26 % of the general population, depending on gender, age and definition of the used criteria [1, 2]. OSA is associated with significant morbidity, such as excessive daytime sleepiness, socially unacceptable snoring and impaired quality of life. Furthermore, if OSA remains untreated, patients are at higher risk of developing cardiovascular diseases [3, 4]. If the apnea-hypopnea index (AHI) is greater than 40 the risk of being involved in traffic accident increases [5, 6].

Bearing this in mind adequate treatment is of key importance. Conservative treatment of OSA consists of lifestyle alterations such as weight reduction, abstinence from alcohol and sedatives and avoidance of supine sleeping position, where appropriate. Continuous positive airway pressure (CPAP), introduced in 1981 by Sullivan, is in many countries regarded as the gold standard in treatment of OSA, with oral device therapy (mandibular reposition appliance, MRA) or surgery in reserve for CPAP failures [7]. Unfortunately CPAP compliance rates are often poor. Weaver and Grunstein report in their review that 29–83 % of patients are non adherent and use their CPAP less than 4 h per night [8].

Since treatment remains indicated in patients with severe OSA with CPAP failure, treatment alternatives are being explored. A variety of site-specific surgical techniques have been developed. The traditional uvulopalatopharyngoplasty (UPPP) or Z-palatopharyngoplasty (ZPP) can be applied in patients with a palatal obstruction [9]. In patients with a base of tongue obstruction site, hyoidthyroidpexia (HTP), radiofrequent ablation of the base of tongue or genioglossus advancement (GA), for example, can be considered.

Traditionally, both subjective (Quality of life, Epworth Sleepiness Scales, etc.) and objective outcomes [polysomnography (PSG) variables] of surgical success are reported in literature.

Success rates of isolated tongue base surgery and of multilevel surgery have been extensively reported and vary between 45 and 62 %, depending on variables such as baseline AHI, BMI, level and configuration of obstruction and on the definition of success used [10–15].

Various PSG parameters such as the AHI or desaturation index (DI) are commonly reported, but rarely attention is paid to the distribution of the variables (AHI for example) in the four sleeping positions, namely the supine, left, right and prone sleep position.

An increasing amount of literature is being published on the role of sleep position in OSA [16–31]. Cartwright was the first to define the current positional OSA (POSA) criteria: an AHI in the worst sleeping position twice or more as compared with the AHI in the other positions [20]. In two studies from Israel and the Netherlands a remarkable steady 56 % of patients have POSA [21, 23, 26]. An additional 30 % of patients have a higher AHI in supine position than in the other positions, but not twice as high.

As early as 1978, Harper and Sauerland [32] suggested that when sleep apnea patients sleep in supine position, the tongue tends to fall backward against the pharyngeal wall, due to gravity. Our group recently reported that base of tongue obstruction or epiglottis obstruction, albeit not statistically significant, is associated with POSA [33].

We therefore question whether sleeping position may play a role in poorly understood successes and failures in sleep surgery, tongue base surgery in particular. We aimed to elucidate the role of sleep position as a confounding factor on AHI and surgical success in tongue base surgery [34].

The aims of this study were to resolve the following:

1. Are unexplained positive or negative outcomes related to variations in percentages of supine sleep position?
2. What is the effect of tongue base surgery on the AHI in supine position in comparison to other sleep positions?
3. Is positional OSA a predictor of surgical outcome?

## Materials and methods

### Patients

We retrospectively reviewed our institutional database of patients diagnosed with OSA and treated with sleep surgery in our hospital from 2004 to 2011. The diagnostic work-up consisted of patient history, physical examination, a full overnight PSG and midazolam or propofol-induced sleep endoscopy to evaluate the site(s) of obstruction and further treatment. In this period (2004–2011) patients were operated on by different surgeons, but all surgical procedures were supervised by one and the same surgeon and thus performed the same way. Patients with moderate to severe OSA and both retrolingual and retropalatal collapse and refusal or non-acceptance of NCPAP treatment were offered multilevel surgical treatment.

In this study we retrospectively included patients with moderate to severe OSA who had undergone a hyoid suspension [35] with or without additional surgical treatment: an uvulopalatopharyngoplasty according to Fujita [36] (in patients with tonsils) or Z-palatoplasty according to Friedman [37] in patients without tonsils and radiofrequent ablation of the base of tongue (RFTB) [38].

### Polysomnography

Polysomnogram recordings were carried out using a digital polygraph system (Embla A10, Broomfield, USA), which recorded the electroencephalogram (FP2-C4/C4-O2), electrooculogram, EKG and submental and anterior tibial electromyogram. Nasal airflow was measured by a pressure sensor and arterial oxygen saturation by finger pulse oximetry. Thoraco-abdominal motion was recorded by straps containing piezoelectric transducers. Snoring was recorded through a piezo snoring sensor. Body position was determined by a position sensor (Sleepsense, St. Charles, USA), which was attached to the midline of the abdominal wall. This sensor differentiated between the upright, left side, right side, prone and supine position. All signals were recorded with DDD (digital sampling, digital filtering, digital storage) recording technology, permitting a sample efficiency of 90 % and a sample rate up to 200 Hz. Storage was done on a PCMCIA flash-card. Data were downloaded to the computer and analyzed by dedicated sleep software (Somnologica, Broomfield, USA) and manually reviewed for analysis by an experienced sleep investigator.

### Definitions

The recommended diagnostic criteria for obstructive sleep apnea syndrome included an apnea hypopnea index (AHI) of five or more and evidence of daytime sleepiness. The AHI was defined as the mean number of apneas and hypopneas per hour during sleep and apnea as a period of 10 s or more with a reduction of oronasal airflow of >90 %. A hypopnea was defined as an episode of more than 30 % airflow reduction of the baseline (calculated from the preceding period of 100 s) during at least 10 s. As per the AASM guidelines AHI thresholds were 5, 15 and 30 events per hour for mild, moderate and severe levels of OSA, respectively [39]. Desaturation index was defined as the number of desaturations ≥4 % for a minimum of 10 s per hour of sleep. Full overnight PSG was repeated 3–4 months postoperatively. Surgical success was defined according to Sher’s criteria: AHI reduction of at least 50 % and AHI reduction to below 20 [40]. When using the AI as an outcome measure the following criteria were applied to define success: reduction by at least 50 % and below a value of 10.

### Statistics

Changes in parameters before and after treatment were tested with a paired Wilcoxon signed rank test. Differences between groups were tested with a χ^2^-test in case of categorical variables and with a Wilcoxon rank sum test in case of continuous variables. The influence of treatment on POSA was tested with the McNemar test for matched pairs. Exact 95 % confidence intervals were calculated for the success proportions and the (overall) response rates. All statistical analyses were performed with SPSS (version 15.0). A *P* value <0.05 was considered to be significant.

## Results

We included 130 patients; patient characteristics are shown in Table 1. 94 patients underwent a combined procedure of base of tongue and palate, from which 72 underwent concurrent radiofrequent thermotherapy of the base of tongue (RFTB). 36 Patients underwent base of tongue surgery alone (HTP), from which 22 underwent concurrent RFTB.

**Table 1:** Baseline characteristics

Variable	Mean ± SD
Age (year)	49.9 ± 9.7
BMI (kg/m^2^)	27.3 ± 2.8
AHI (/h)	36.7 ± 14.4
AHI supine (/h)	51.2 ± 24.8
AHI supine (%)	37.4 ± 24.7
Ratio (male:female)	9:1

No significant differences in AHI, supine AHI, non-supine AHI, percentage of supine sleep position, total sleep time, arousal index and awakenings were found between the different surgical groups when divided into solely base of tongue surgery (HTP either with or without concurrent RFTB) and combined base of tongue and palate surgery (with either UPPP or ZPP) (Table 2).

**Table 2:** Mean values of AHI and AHI in different positions, before and after surgery for different groups of patients

	Mean (before)	Mean (after)	*P* value (within)	Mean (before)	Mean (after)	*P* value (within)	*P* value (between)
AHI successful^a^	Yes (*n* = 49)			No (*n* = 81)			
AHI	35.8	9.5	**<0.001**	37.2	34.5	0.099	**<0.001**
AHI supine	49.1	18.9	**<0.001**	52.5	51.6	0.749	**<0.001**
AHI non supine	23.1	5.1	**<0.001**	27.7	23.9	0.052	**<0.001**
AHI prone	4.9	3.5	0.647	15.0	8.1	**0.033**	0.234
AHI left	18.7	4.9	**<0.001**	26.8	20.3	**0.004**	0.077
AHI right	15.9	4.8	**<0.001**	24.2	19.9	0.137	0.061
% supine sleep position	42.8	36.9	0.126	34.2	37.9	0.124	**0.024**
AI successful^b^	Yes (*n* = 54)			No (*n* = 76)			
AI	20.0	2.7	**<0.001**	21.0	21.9	0.376	**<0.001**
AHI	34.4	13.1	**<0.001**	38.3	33.9	**0.026**	**<0.001**
AHI supine	51.8	24.1	**<0.001**	51.5	50.5	0.792	**<0.001**
AHI non supine	22.7	8.7	**<0.001**	28.2	22.8	**0.011**	**0.003**
AHI prone	10.4	3.4	0.154	11.9	8.5	0.161	0.922
AHI left	21.9	8.3	**<0.001**	25.4	19.1	**0.006**	**0.029**
AHI right	18.5	7.5	**0.001**	23.3	19.2	0.098	0.141
% supine sleep position	36.8	37.7	0.679	38.4	37.7	0.886	0.720
AHI or AI successful	Yes (*n* = 65)			No (*n* = 65)			
AHI	34.8	13.1	**<0.001**	38.5	37.1	0.501	**<0.001**
AHI supine	49.8	23.8	**<0.001**	52.7	54.8	0.498	**<0.001**
AHI non supine	23.6	8.4	**<0.001**	28.3	25.2	0.152	**<0.001**
AHI prone	9.4	3.5	0.115	12.9	9.2	0.197	0.808
AHI left	20.8	8.2	**<0.001**	26.7	20.9	**0.023**	**0.027**
AHI right	18.0	6.9	**<0.001**	24.2	21.4	0.258	0.071
% supine sleep position	38.2	37.2	0.838	36.7	37.9	0.575	0.565
Positional OSA^c^	Yes (*n* = 70)			No (*n* = 60)			
AHI	32.7	23.7	**<0.001**	41.3	26.6	**<0.001**	**0.044**
AHI supine	57.9	42.2	**<0.001**	43.5	35.6	0.132	0.107
AHI non supine	14.9	13.3	0.106	38.8	20.8	**<0.001**	**<0.001**
AHI prone	4.4	6.3	0.793	19.1	6.4	**0.002**	**0.039**
AHI left	16.0	11.6	0.159	32.8	17.9	**<0.001**	**<0.001**
AHI right	12.8	13.0	0.501	30.8	15.6	**<0.001**	**0.001**
% supine sleep position	43.9	43.9	0.654	29.9	30.1	0.853	0.642
Treatment	HTP/HTP + RFTB (*n* = 36)			HTP + UPPP/ZPP (*n* = 94)			
AHI	26.7	16.4	**<0.001**	38.4	26.5	**<0.001**	0.744
AHI supine	41.4	30.4	0.059	52.9	40.7	**<0.001**	0.837
AHI non supine	19.8	8.5	**0.001**	27.0	18.2	**<0.001**	0.676
AHI prone	9.6	0.5	**0.043**	11.4	7.4	0.133	0.275
AHI left	15.5	10.8	0.055	25.2	15.2	**<0.001**	0.474
AHI right	19.0	8.5	**0.010**	21.5	15.1	**0.008**	0.365
% supine sleep position	34.2	42.1	0.260	38.0	36.8	0.750	0.162

Boldfaced values are the significant differences before and after treatment within the groups or between the groups

^a^Reduction in AHI of at least 50 % and to below 20

^b^Reduction in AI of at least 50 % and to below 10

^c^AHI supine/AHI non supine >2

The mean AHI of all 130 patients decreased significantly from 36.7 (range 9.0–100.9) to 25.1 (*P* < 0.001). AHI in supine position decreased significantly from 51.2 to 39.3 (*P* < 0.001). AHI in left position decreased significantly from 23.7 to 11.2 (*P* < 0.001). AHI in right position decreased significantly from 21.1 to 14.2 (*P* < 0.001). AHI in prone position decreased significantly from 11.2 to 6.4 (*P* < 0.001).

A successful reduction in AHI, according to Sher’s critera was seen in 49 patients (CI 29.3–46.6 %) and in AI in 54 patients (CI 33.2–50.9 %). Half of the patients (CI 41.1–58.9 %) either had a successful reduction in AHI or in AI.

In general, patients who had a successful reduction in AHI slept less often in supine position after treatment than before treatment compared with the patients who did not have a successful reduction in AHI (*P* = 0.024). The difference between the percentage of total sleep time (TST) in supine position before and after surgery was not significant within each group (*P* = 0.126 and 0.124 for both groups, respectively). The mean difference in AHI before and after treatment was 26.3/h in the group of patients with a successful reduction in AHI, which is significantly higher than the mean difference in the other group (*P* < 0.001). The mean AHI in supine position decreased by 30.2/h in the first group, again significantly higher than the difference in the other group (*P* < 0.001). The differences in AHI, AHI in supine position and AHI in non-supine position before and after treatment were all significant for the patients with successful AHI reduction (all *P* < 0.001), and not for the patients in the non-successful group (*P* = 0.099 total AHI, *P* = 0.749 AHI supine, *P* = 0.052 AHI non supine). The AHI in left or right position decreased significantly for the successful group, whereas AHI in prone position did not. In the unsuccessfully treated group, the AHI in prone and left position did decrease significantly after surgery (Table 2). Total sleep time, arousal index and number of awakenings did not change significantly after surgery (data not shown here).

Seventy patients suffered from POSA pre-operatively. Within this group the total AHI decreased significantly from 32.7 to 23.7 (*P* < 0.001), and the AHI in supine position decreased significantly from 57.9 to 42.2 (*P* < 0.001). The percentage of patients that slept in supine position did not change significantly after treatment (*P* = 0.654). In 34 of the 70 position-dependent patients the treatment was successful (CI 36.4–60.1 %) (Table 3). The other 60 pre-operative non-POSA patients had a significantly lower AHI following surgery (*P* < 0.001). A significant decrease of the AHI was seen in all positions except the AHI in supine position. The difference in supine AHI before and after treatment between the POSA and non-POSA groups was not significant (*P* = 0.107), while the AHI decreased more for the non-POSA patients (*P* = 0.044) (Table 2). Furthermore, in the non-POSA patients the post-operative decrease in non-supine, prone, left and right AHI was significant when compared with these parameters in the POSA patients (*P* < 0.001, *P* = 0.039, *P* < 0.001, *P* = 0.001).

**Table 3:** Success rates split for different outcomes of positional OSA

	Percentage	95 % CI	Percentage	95 % CI	*P* value
Positional OSA^a^	Yes (*n* = 70)		No (*n* = 60)		
AHI successful	35.7	24.6–48.1	40.0	27.6–53.5	0.615
AI successful	42.9	31.1–55.3	40.7	28.1–54.3	0.803
AHI or AI successful	48.6	36.4–60.1	51.7	38.4–64.7	0.725

The *P* values denote the difference in these rates between the two groups

^a^AHI supine/AHI non supine >2

**Fig. 1:**
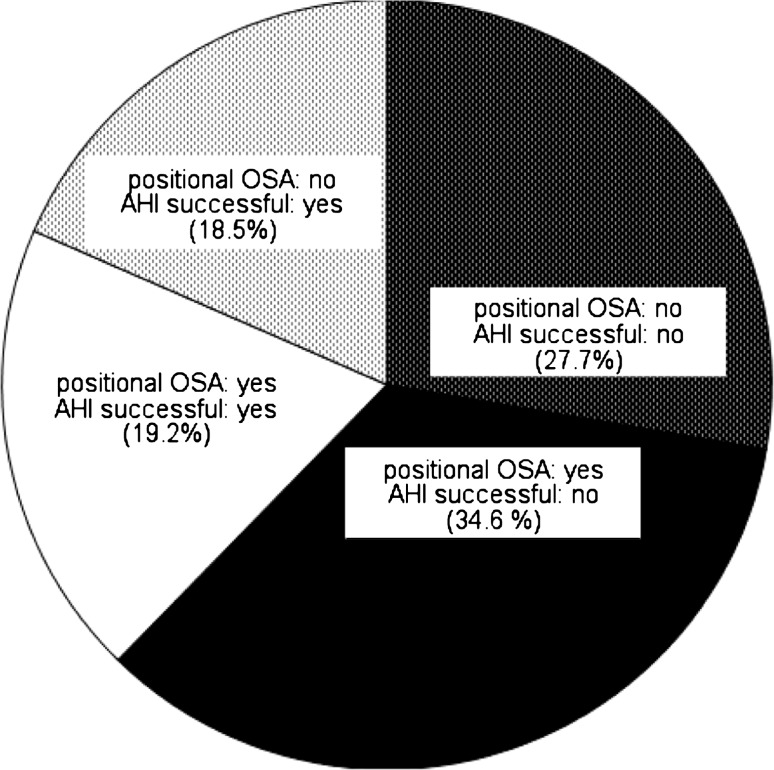
Distribution of surgical success amongst POSA and non-POSA patients

Surgery was not more successful in the group with position-dependent patients than in the other group (*P* = 0.615) (Fig. 1). Most positional patients remained positional after surgery and most non-positional patients remained non-positional (72.9 and 57.6 %, *P* = 0.451) (Table 4).

**Table 4:** Effect of surgery on position-dependency

	Positional OSA^a^	Post treatment				*P* value
		No		Yes		
		*n*	%	*n*	%	
Pre treatment	No	34	57.6	25	42.4	0.451
	Yes	19	27.1	51	72.9	

^a^AHI supine/AHI non supine >2

## Discussion

The present study is the first which looks into the relation between tongue base surgery either with or without concurrent palate surgery and sleep position.

Our study population did not solely consist of isolated base of tongue surgery patients but also included patients who concurrently underwent palate surgery. Ideally we would have studied isolated base of tongue surgery. But most patients we diagnostically worked up for HTP suffered from multilevel obstruction. Although we compared the isolated base of tongue surgery group with the combined base of tongue and palate surgery group and found no significant differences between the groups, this is a limitation of our study.

To our best knowledge only three earlier studies have been published on the effect of sleep position on outcomes of palate surgery (UPPP) [34, 41, 43]. So far, no papers have been published on the combination approach of sleep surgery and positional therapy (PT).

The overall success rate and overall response rate of this series of HTP/tongue base surgery with or without concomitant palatal surgery in patients with moderate to severe OSA and CPAP failure are 38 and 60 %, respectively, which is in the low-normal range compared with previously reported series [10–15]. Improvement of treatment outcome is mandatory if treatment intent is “salvage” in CPAP failures.

It is a clinical reality in sleep surgery that remarkable differences in outcome can occur amongst patients with comparable pre-operative AHI, BMI, clinical findings such as tongue size, tonsil size and drug-induced sleep endoscopy (DISE) findings [33, 43]. We took a closer look to evaluate whether discrepancies between expected and actual outcome could be explained by changes in body position before and after treatment. For this reason the patients in the present series were divided into positional and non-positional groups.

In general, in both positional and non-positional patients, the percentage supine sleep position remained remarkably constant after surgery and remarkable successes or failures could not be explained by considerable changes in percentage supine position. In conclusion, surgery did not influence patient’s position-dependency (Table 4).

Hyoid suspension is traditionally thought to exert its effect by increasing the retrolingual airway space. We hypothesised therefore that in successful surgery, more outspoken decreases in AHI would be found in the supine position, than in other sleep positions.

Our results show that HTP did not have better effect on the supine AHI in comparison with the AHI in other sleeping position components. When surgery was either successful or non-successful, the reduction in AHI was uniform in all sleeping positions.

Earlier Stuck et al*.* reported that MRI studies do not show enlargement of the retrolingual airway space following HTP. These authors concluded that the effect of HTP was in increased general stabilization of the upper airway, not an enlargement of the retrolingual airway [44]. Our present findings provide further support for this concept. However, our follow-up was relatively short (3–4 months). Further research, to evaluate long term results is ongoing.

POSA occurs in 56 % [26] of OSA patients. PT as treatment for POSA is gaining momentum [30]. After surgical failure in positional patients, a further decrease of the AHI can theoretically be accomplished by prevention of the supine sleep position. This leads to the concept of multimodality treatment. Theoretically, in POSA, multilevel surgery with PT would achieve better results than surgery or PT alone. This is in concordance with earlier research papers in palate surgery. Katsantonis et al. [41] studied the effect of UPPP on sleep posture and differences in uvulopalatopharyngoplasty (UPPP) results in various sleep positions in a small series of 17 patients. They found that following UPPP, the AHI significantly improved in the lateral position. They also found that during sleep in a supine position, the AHI did not show significant improvement. They conclude that UPPP enhances the position effect on OSA because it readily eliminates obstructive events in the lateral sleep position. In other words, the difference in AHI in supine and non-supine positions are more pronounced postoperatively. They are of opinion that additional positional therapy could significantly improve response to treatment with UPPP. Lee et al. [34] studied the effect of sleep position on surgical outcomes as well. They studied 69 consecutive patients who underwent a UPPP. After categorizing the patients into four groups according to the change in AHI after surgery, they found that the failure group had a higher proportion of supine position dependency than any other group. In a second paper published by the same group, results show that UPPP is a successful treatment for obstructive events occurring in the lateral sleep position, especially in patients without positional dependency [42]. A suggestion is made that patients who have become position-dependent may benefit from positional therapy after UPPP.

Until recently, PT consisted of the “tennis ball technique”. A variety of tennis balls, squash balls, shark fins, special pajamas and vests all had the same concept of a bulky mass worn on the back. All these devices have in common that they are not comfortable, disrupt sleep architecture and the long-term compliance is a disappointing 10 % [45]. A recent paper by our group for the first time showed that a small buzzing device worn in the neck can prevent supine sleeping position without disrupting sleep [30].

## Conclusion

The difference between the percentage of total sleep time in supine position before and after surgery was not significant. The differences in AHI, AHI in supine position and AHI in non-supine position before and after treatment were all significant for the patients with successful AHI reduction (all *P* < 0.001), and not for those patients in the non-successful group (*P* = 0.099 total AHI, *P* = 0.749 AHI supine, *P* = 0.052 AHI non supine).

Isolated tongue base or multilevel surgery was as successful on the supine AHI as it was on the AHI in other sleeping positions.

Surgery was not more successful in the group with position-dependent patients as compared with the non-position-dependent patients (*P* = 0.615). Successful and non-successful surgical results could not be explained by variations in percentages of supine sleep position.

Surgery was not more successful in the group with position-dependent patients than in the non-position-dependent group (*P* = 0.615).

From this retrospective analysis we conclude that sleep position is not a confounding factor on surgical outcomes in tongue base surgery.

The results of base of tongue or multilevel surgery in position-dependent OSA patients leave room for improvement, possibly through positional therapy. Further research on the combined effect of multilevel surgery and positional therapy is ongoing.

## References

[1] Kryger MH (2000). Diagnosis and management of sleep apnea syndrome. Clin Cornerstone.

[2] Young T, Hutton R, Finn L, Baddr S, Palta M (1996). The gender basis in sleep apnea diagnosis: are women missed because they have different symptoms?. Arch Intern Med.

[3] Young T, Finn L, Peppard PE (2008). Sleep disordered breathing and mortality: eighteen-year follow-up of the Wisconsin sleep cohort. Sleep.

[4] Redline S, Yenokyan G, Gottlieb DJ (2010). Obstructive sleep apnea-hypopnea and incident stroke: the sleep heart health study. Am J Respir Crit Care Med.

[5] Findley LJ, Weiss JW, Jabour ER (1991). Drivers with untreated sleep apnea. A cause of death and serious injury. Arch Intern Med.

[6] Smolensky MH, Di Milia L, Ohayon MM, Philip P (2011). Sleep disorders, medical conditions, and road accident risk. Accid Anal Prev.

[7] Randerath WJ, Verbraecken J, Andreas S (2011). European Respiratory Society task force on non-CPAP therapies in sleep apnoea. Non-CPAP therapies in obstructive sleep apnoea. Eur Respir J.

[8] Weaver TE, Grunstein RR (2008). Adherence to continuous positive airway pressure therapy: the challenge to effective treatment. Proc Am Thorac Soc.

[9] Woodson BT (2008). Structural effectiveness of pharyngeal sleep apnea surgery. Sleep Med Rev.

[10] Kezirian EJ, Goldberg AN (2006). Hypopharyngeal surgery in obstructive sleep apnea: an evidence-based medicine review. Arch Otolaryngol Head Neck Surg.

[11] Baisch A, Maurer JT, Hörmann K (2006). The effect of hyoid suspension in a multilevel surgery concept for obstructive sleep apnea. Otolaryngol Head Neck Surg.

[12] Richard W, Kox D, den Herder C, van Tinteren H, de Vries N (2007). One stage multilevel surgery (uvulopalatopharyngoplasty, hyoid suspension, radiofrequent ablation of the tongue base with/without genioglossus advancement), in obstructive sleep apnea syndrome. Eur Arch Otorhinolaryngol.

[13] Benazzo M, Pagella F, Matti E (2008). Hyoidthyroidpexia as a treatment in multilevel surgery for obstructive sleep apnea. Acta Otolaryngol.

[14] Van den Broek E, Richard W, van Tinteren H, de Vries N (2008). UPPP combined with radiofrequency thermotherapy of the tongue base for the treatment of obstructive sleep apnea syndrome. Eur Arch Otorhinolaryngol.

[15] Yi HL, Sun XQ, Chen B (2011). Z-palatopharyngoplasty plus genioglossus advancement and hyoid suspension for obstructive sleep apnea hypopnea syndrome. Otolaryngol Head Neck Surg.

[16] Kavey NB, Blitzer A, Gidro-Frank S, Korstanje K (1985). Sleeping position and sleep apnea syndrome. Am J Otolaryngol.

[17] Cartwright RD (1984). Effect of sleep position on sleep apnea severity. Sleep.

[18] Chaudhary BA, Chaudhary TK, Kolbeck RC, Harmon JD, Speir WA (1986). Therapeutic effect of posture in sleep apnea. South Med J.

[19] George CF, Millar TW, Kryger MH (1988). Sleep apnea and body position during sleep. Sleep.

[20] Cartwright RD, Diaz F, Lloyd S (1991). The effect of sleep posture and sleep stage on apnea frequency. Sleep.

[21] Oksenberg A, Silverberg DS, Arons E, Radwan H (1997). Positional vs nonpositional obstructive sleep apnea patients. Chest.

[22] Itasaka Y, Miyazaki S, Ishikawa K, Togawa K (2000). The influence of sleep position and obesity on sleep apnea. Psychiatry Clin Neurosci.

[23] Oksenberg A, Khamaysi I, Silverberg DS, Tarasiuk A (2000). Association of body position with severity of apneic events in patients with severe nonpositional obstructive sleep apnea. Chest.

[24] Akita Y, Kawakatsu K, Hattori C, Hattori H, Suzuki K, Nishimura T (2003). Posture of patients with sleep apnea during sleep. Acta Otolaryngol Suppl.

[25] Mador MJ, Kufel TJ, Magalang UJ, Rajesh SK, Watwe V, Grant BJ (2005). Prevalence of positional sleep apnea in patients undergoing polysomnography. Chest.

[26] Richard W, Kox D, den Herder C, Laman M, van Tinteren H, de Vries N (2006). The role of sleeping position in obstructive sleep apnea. Eur Arch Otorhinolaryngol.

[27] Permut I, Diaz-Abad M, Chatila W, Crocetti J, Gaughan JP, D’ Alonzo GE, Krachman SL (2010). Comparison of positional therapy to CPAP in patients with positional obstructive sleep apnea. J Clin Sleep Med.

[28] van Kesteren ER, van Maanen JP, Hilgevoord AA, Laman DM, de Vries N (2011). Quantitative effects of trunk and head position on the apnea hypopnea index in obstructive sleep apnea. Sleep.

[29] Schwab RJ (2011). A quantum advance in PSG recordings: the importance of head position in mediating the AHI. Sleep.

[30] van Maanen JP, Richard W, van Kesteren ER, Ravesloot MJL, Laman DM, Hilgevoord AAJ, de Vries N (2011) Evaluation of a new simple treatment for positional sleep apnea patients. J Sleep Res [Epub ahead of print] 10.1111/j.1365-2869.2011.00974.x 22017727

[31] Ravesloot MJL, Frank MH, Van Maanen JP, Dun L, Verhagen E, de Lange J, de Vries N. Positional OSA (2012) How often is it clinical relevant and how often is treatment indicated. A proposal for a new classification system, based on its clinical significance (submitted)

[32] Harper R, Sauerland E (1978) The role of the tongue in sleep apnea. In: Guilleminault C, Dement W (eds) Sleep apnea syndromes. pp 219–34

[33] Ravesloot MJL, de Vries N (2011). 100 consecutive patients undergoing drug-induced sleep endoscopy—results and evaluation. Laryngoscope.

[34] Lee CH, Shin HW, Han DH (2009). The implication of sleep position in the evaluation of surgical outcomes in obstructive sleep apnea. Otol Head Neck Surg.

[35] Riley RW, Powell NB, Guilleminault C (1994). Obstructive sleep apnea and the hyoid: a revised surgical procedure. Otolaryngol Head Neck Surg.

[36] Fujita S, Conway W, Zorick F, Roth T (1981). Surgical corrections of anatomical abnormalities in obstructive sleep apnea syndrome: uvulopalatopharyngoplasty. Otolaryngol Head Neck Surg.

[37] Friedman M, Ibrahim HZ, Vidyasagar R, Pomeranz J, Joseph NJ (2004). Z-palatoplasty (ZPP): a technique for patients without tonsils. Otolaryngol Head Neck Surg.

[38] Stuck BA, Köpke J, Maurer JT (2003). Lesion formation in radiofrequency surgery of the tongue base. Laryngoscope.

[39] (1999) Sleep-related breathing disorders in adults: recommendations for syndrome definition and measurement techniques in clinical research. The Report of an American Academy of Sleep Medicine Task Force. Sleep 22:667–89 10450601

[40] Sher AE, Schechtman KB, Piccirillo JF (1996). The efficacy of surgical modifications of the upper airway in adults with obstructive sleep apnea syndrome. Sleep.

[41] Katsantonis GP, Miyazaki S, Walsh J (1990). Effects of uvulopalatopharyngoplasty on sleep architecture and patterns of obstructed breathing. Laryngoscope.

[42] Lee CH, Kim SW, Han K (2011). Effect of uvulopalatopharyngoplasty on positional dependency in obstructive sleep apnea. Arch Otolaryngol Head Neck Surg.

[43] Kezirian EJ, Hohenhorst W, de Vries N (2011). Drug-induced sleep endoscopy: the VOTE classification. Eur Arch Otorhinolaryngol.

[44] Stuck BA, Neff W, Hörmann K (2005). Anatomic changes after hyoid suspension for obstructive sleep apnea: an MRI study. Otolaryngol Head Neck Surg.

[45] Oksenberg A, Silverberg D, Offenbach D, Arons E (2006). Positional therapy for obstructive sleep apnea patients: a 6-month follow-up study. Laryngoscope.

